# Stereotypical Escape Behavior in *Caenorhabditis elegans* Allows Quantification of Effective Heat Stimulus Level

**DOI:** 10.1371/journal.pcbi.1005262

**Published:** 2016-12-27

**Authors:** Kawai Leung, Aylia Mohammadi, William S. Ryu, Ilya Nemenman

**Affiliations:** 1 Department of Physics, Emory University, Atlanta, Georgia, United States of America; 2 Department of Physics, University of Toronto, Toronto, Ontario, Canada; 3 Donnelly Centre, University of Toronto, Toronto, Ontario, Canada; 4 Department of Biology, Emory University, Atlanta, Georgia, United States of America; Northeastern University, UNITED STATES

## Abstract

A goal of many sensorimotor studies is to quantify the stimulus-behavioral response relation for specific organisms and specific sensory stimuli. This is especially important to do in the context of painful stimuli since most animals in these studies cannot easily communicate to us their perceived levels of such noxious stimuli. Thus progress on studies of nociception and pain-like responses in animal models depends crucially on our ability to quantitatively and objectively infer the sensed levels of these stimuli from animal behaviors. Here we develop a quantitative model to infer the perceived level of heat stimulus from the stereotyped escape response of individual nematodes *Caenorhabditis elegans* stimulated by an IR laser. The model provides a method for quantification of analgesic-like effects of chemical stimuli or genetic mutations in *C. elegans*. We test ibuprofen-treated worms and a TRPV (transient receptor potential) mutant, and we show that the perception of heat stimuli for the ibuprofen treated worms is lower than the wild-type. At the same time, our model shows that the mutant changes the worm’s behavior beyond affecting the thermal sensory system. Finally, we determine the stimulus level that best distinguishes the analgesic-like effects and the minimum number of worms that allow for a statistically significant identification of these effects.

## Author Summary

A doctor assesses pain by asking her patient to “rate your pain on the scale of 1 to 10.” She may then prescribe some drugs and later ask the question again to see if they worked. New drugs are often developed using animal models, but we cannot ask an animal, especially a small invertebrate animal, to rate, similarly, the strength of its perceived noxious stimulus. In this paper, we successfully develop computational tools that read the “body language” of a roundworm *C. elegans* to estimate the strength of the heat stimulus that it experiences. Unlike previous attempts that have focused on ad hoc selected components of the overall behavior, our approach is based on quantifying the complete time series of the escape behavior, which we show to be captured by a behavioral “template” that scales in response to the stimulus strength. The existence of this template allows us to solve one of the hard questions in pain research: disambiguating analgesic-like effects of drugs or genetic perturbations from their other effects on animal behavior.

## Introduction

A *grand goal* in understanding sensory systems is to predict the behavioral response of an organism to specific sensory stimuli; or, conversely, to infer the sensory stimuli from measurements of the behavioral response. Such a goal requires careful experimental design, precise control of the sensory inputs, and quantification of the behavioral outputs. While sensory stimuli can be carefully quantified and applied, the quantification of the behavioral output and its relationship to the stimulus is non-trivial because the behavior is often complicated and not well defined. In this work we address this grand goal in the context of studies of pain, or nociception (i. e., sensing of noxious stimuli, which damage or threaten to damage normal tissues), where behavioral quantification is especially hard. (Note that, in this paper, we use nomenclature developed by the International Association for the Study of Pain, http://www.iasp-pain.org/Taxonomy.)

Pain studies on human subjects are difficult because of ethical constraints, difficulties in quantifying a psychophysical response, and subjectivity in self-reporting [1]. Partial conservation of molecular mechanisms of nociception among many different species [2–4] allows to solve some of these problems by using animal models. However, then the grand goal of quantifying the behavior and relating it to the stimulus becomes even harder: animal subjects cannot communicate their perceived noxious levels to us in an obvious fashion. Thus progress in using animal models depends crucially on our ability to quantitatively and objectively infer the perceived level of noxious stimuli from animal behavior.

Historically, studies of nociception primarily used mammalian models [5, 6]. For rodents, the tail flick test [7], the hot-plate test [8, 9] and the Hargreaves’ method [10] correlate pain perception with the reaction latency of different body parts to noxious stimuli. For larger animals such as canines [11] and primates [12, 13], similar nociceptive assays have also been developed. In recent years, new approaches started incorporating facial expressions in nociception quantification [14–16]. Although these mammalian models are extensively studied, several drawbacks hinder their use. First, ethical issues and risks arise for certain experiments. Second, compared to invertebrates, vertebrate subjects require more time and resources to maintain. Therefore, much effort has been devoted to investigations of the possibility of using invertebrate models in nociception research [3, 17]. In experiments involving *Drosophila* larva, measures such as the response percentage of the total population [18, 19] and the time to response [20] have been used to investigate changes in the ability of the animals to sense noxious stimuli. In experiments on *Caenorhabditis elegans*, behavioral features such as the turning rate [2] and the percentage of escape response [21] have been used to characterize nociception.

All of these models share some common problems. First, the nociceptive assays focus on one particular coarse behavioral feature of the subject, such as avoidance behavior, orientation, or turning rate. Such features are selected in an ad hoc fashion, subject to a particular design of an experiment. This makes it difficult to compare results across different labs and experiments. Further, this does not solve the grand goal of quantifying the full stimulus-behavior relation, and thus the behavior may be providing additional information about the perceived noxious stimulus level that is not being captured by the coarse measures. Second, some assays report measurements as a percentage of a population, so that these measurements cannot be made for individuals. To overcome these problems, an ideal assay would infer a perceived noxious stimulus level of an individual animal on a continuous scale, using comprehensive, objective measurements of its behavioral profile.

Solving the grand goal of quantifying the stimulus-behavior relation in the context of pain studies would allow one to use the assays to calibrate the perceived noxious stimulus level, and maybe even reductions in such levels due to analgesic-like effects of drugs, or mutations in the nociceptive pathways. At the same time, drugs or mutations can affect the motor response, rather than the nociception per se. Thus traditional pain assays mentioned above may convolve the perceived noxious stimulus reduction, if any, with behavioral changes. For example, a mutant defective for turning behavior will register a strong reduction in the turning rate, but it would be a mistake to interpret this as a reduction in nociception. Such concerns are very real, as is illustrated by a known fact that opioids can cause large behavioral changes [22]. To attribute a behavioral response difference to reduced nociception and not to motor changes, the response must be stereotyped and reflexive, which is often the case [6]. Further, only the response amplitude or frequency, but not the detailed temporal structure, should change in response to a drug or a mutation. Establishing the stability of the stereotypic response pattern requires solving the grand goal: analysis of the *entire stimulus-triggered response behavior*, rather than of its few selected features, as is done by most behavioral assays.

In this work, we address these issues in the context of the nematode *C. elegans*, solving the grand goal in the context of its heat-evoked escape behavior, and hence developing the worm further as an animal model system for nociception research. The worm is a great model organism for such studies for a number of reasons. First, the behavioral dynamics of freely moving *C. elegans* is intrinsically low dimensional [23]. This makes quantification of its behavioral response relatively straightforward, providing an opportunity to use the entire motile behavior as a basis for assays. Second, the worms show a noxious response to a wide range of sensations including certain types of chemical [24, 25], mechanical [26, 27], and thermal [28, 29] stimuli, and such a nociceptive response is different from and is transduced independently of the related taxis behaviors [30–34]. Third, at the molecular level, many details of heat nociception in the worm may be similar to vertebrate animals [3]. Fourth, there are powerful genetic and optical tools to reveal mechanisms of nociception in *C. elegans*. Finally, the low cost, small size, and absence of ethical constraints make the animal amenable to large scale pharmacological screens for new human analgesics [35].

We present combined experimental and modeling studies that show that the entire temporal behavioral profile during the heat-evoked escape response in *C. elegans* is highly stereotypical, with the frequency of the escape response and the amplitude of the escape velocity profile scaling with the stimulus level. By verifying the ability of the behavioral template to capture the response following a heat stimulus, the model we develop distinguishes changes in the sensory system from changes to the motor program. When a change is attributed to the sensory system, the model can infer the reduction in the perceived heat stimulus level following pharmacological or genetic treatments from the behavior of an individual worm. This quantification requires only about 60 worms to show statistically significant perceived stimulus reduction for a common human analgesic, and its statistical power quickly improves with an increasing number of subjects.

Overall, this solution of the grand goal in the worm heat-evoked escape context suggests that, for *C. elegans*, it is possible to disambiguate perturbations to the sensory system from other perturbations affecting motility, and to quantify the reduction in the perceived heat stimulus from behavioral data. Combined with the previous evidence, this bodes well for further establishment of the worm as a model system for pain research. However, we stress that the differences between the worm and the human are so large that we do not want to overstate the importance of a *C. elegans* nociception model in the study of human health. Our analysis may be useful in the future for identifying molecular mechanisms of nociception (which may be similar to those leading to pain in humans), or identification of drugs that affect them. Some of these drugs may even work in humans, but there is no reason to believe that pain (and especially its emotional component) occurs in *C. elegans*, or that drugs that affect nociception actually produce analgesic effect in worms. Thus the main contribution of our work is in addressing the *grand goal*, namely in quantitative characterization of regularities of complete heat-evoked escape behavior *C. elegans* on a single-subject level, and analysis of changes in these regularities under different pharmacological and genetic treatments, rather than potential applications of our findings to future discovery of new human analgesics.

The paper is organized as follows. First we discuss the structure of the dataset and the model. Then we evaluate the performance of the model and discuss the stereotypical behavior we discovered. Further, we use the model to infer the heat stimulus level of worms in three different conditions: wild-type untreated, wild-type treated with ibuprofen, and a mutant with defects in thermal nociception. We argue that, while effects of the ibuprofen treatment can be attributed largely to reduced nociception, the mutant’s response shows changes to the behavior beyond nociceptive effects. Finally, we use the statistical model to discuss how the nociception experiments should be designed to achieve the highest statistical power to quantify the analgesic-like effects.

## Results

We aim to infer the perceived heat stimulus level from the temporal dynamics of the worm response. The heat stimulation is administered using an infrared laser while the worm crawls on an agar plate. The worm motion is captured by video microscopy and analyzed using custom image analysis software. The worm postures are very stereotypical, adding up to simple sinusoidal motions forwards or backwards, and to turns [23]. Thus without much loss of the statistical power, we characterize the entire escape behavior of the animal by a time series of its center of mass velocity (Materials and Methods). Our task is then to verify if such responses are stereotypical, scaling in frequency and amplitude with the applied laser current. If they are stereotypical and thus can be used to characterize the perceived stimulus level, the next task is to infer the applied laser current from the velocity data.

**Fig 1:**
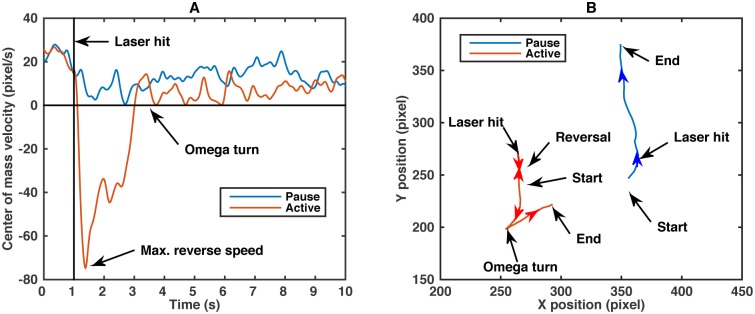
Typical center-of-mass heat-evoked escape responses in *C. elegans*. (A) Typical center-of-mass trajectory of two wild-type worms in a paused and an active state (see below for the detailed discussion of the two states). The infrared laser was directed at each worm at *t* = 1 s. The paused state is characterized by the near zero velocity after the laser stimulus. (B) Actual trajectories of these two worms. Worm changes its direction of motion in two ways: in a “reversal”, it stops and backtracks along its previous path; in an “omega” turn the worm’s head curls back and crosses the tail, setting then a new direction of forward motion.

For each heat stimulus trial a random worm is selected on the plate and its motion is sampled at 60 Hz for 15 s. An infrared laser pulse with a randomly chosen current between 0 to 200 mA and a duration of 0.1 s is then directed to the head of the worm 1 s after the start of the video recording. The worm’s center of mass motion in a typical trial consists of a forward motion before the stimulus, a stop and/or backward motion after the stimulus, then followed by an “omega” turn, after which the worm emerges with a forward motion in a different direction (Fig 1). This is a typical response to many noxious stimuli in *C. elegans*, and not just to heat stimulus [29] (stereotyped, typical escape responses also exist in other animals [36, 37]). Further, previous research has shown that, even though the maximum temperature change in this assay is rather small (∼1 − 2°C), the escape behavior is significantly different from the more commonly studied thermotactic behavior, and is mediated by different neural and genetic pathways [28, 29, 38].

To understand the effects of pharmacological and genetic interventions, we collected three distinct datasets. In the first, the stimulus was applied to wild-type worms (N2). In the second, the wild-type worms were pre-treated with an ibuprofen solution (Materials and Methods), which we surprisingly discovered to affect the heat-evoked response, after screening some common human analgesics. Since ibuprofen’s molecular mechanism of action in *C. elegans* is unclear, we refer to this as an analgesic-like treatment throughout the paper. In the third, we applied the stimulus to an untreated triple mutant (ocr-2(ak47) osm-9(ky10) IV; ocr-1(ak46)), which is widely used in *C. elegans* nociception studies; it has one of the strongest effects on reduction of heat-evoked nociceptive response [38]. Hereafter we refer to these datasets as “control”, “ibuprofen”, and “mutant”, respectively. We chose not to explore hyperalgesic treatments for this study. We collectively refer to ibuprofen and mutant worms as “treated” worms. Forward motion in all three data sets was similar (typical forward velocity of 13 ± 9, 9 ± 7, 13 ± 10 pixel/s respectively, where the error denoted the standard deviation of the velocity distribution), so that there are no drastic defects in motility.

### Statistical model of the heat-evoked escape

**Fig 2:**
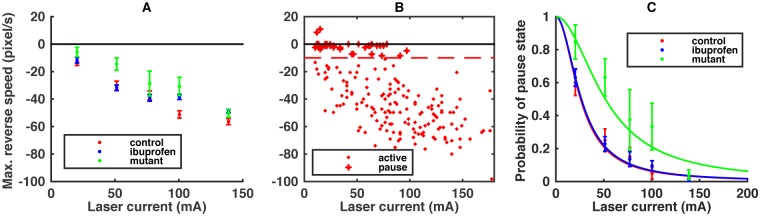
Global characteristics of the heat-evoked escape. (A) The mean (± s. e. m.) of the maximum reverse speed for the three worm types is plotted against the stimulus laser current, partitioned into five bins. Negative values correspond to backward motion. (B) Maximum reverse speeds for individual control worms. We define an active worm (dot) as having the maximum reverse speed of over 10 pixels/s (about 0.2 body length per second); otherwise the worm is paused (plus sign). (C) Probability of the paused state vs. the laser current. Dots represent the actual data (± s. e. m.), and lines are the fitted model. The datasets are divided into equally sampled bins for each worm type.

For presentation purposes, we bin the laser current of the heat stimulus into five distinct levels (bins), defined to have an equal number of control worms in each bin (40 per bin). The maximum reverse escape velocity is indistinguishable among all three worm types for the largest stimulus level, indicating no gross defects to motility or noxious response. At the same time, the velocity at smaller stimuli levels shows substantial differences (*Z* scores of up to 3.8) between the control and the treated worms, especially in the vicinity of ∼100 mA laser stimulation (Fig 2A). Two-way ANOVA shows the difference between the ibuprofen and the control worms at *p* = 0.021 and between the mutant and the control at *p* = 1.5 ⋅ 10^−5^ across all five laser current groups. Mutant worms are especially different from the control over a wider stimulus range. However, as discussed above, it is unclear if such simple observed behavioral differences are indicative of the reduction of the perceived stimulus level or of other changes to the motor response. Furthermore, there might be additional changes in the detailed temporal structure of the heat-evoked escape dynamics, which would not be captured by simple statistics, such as the maximum reverse speed.

To address this, instead of subjectively segmenting the complex escape behavior or choosing *ad hoc* metrics of the worm’s movement, we choose to infer the applied stimulus strength from a comprehensive model of the entire worm’s response velocity profile. Due to the considerable randomness and individual variability of responses, we choose to model them probabilistically (see [39] for another example of Bayesian probabilistic modeling of nociception). Thus we are interested in estimating *P*(*I*|**v**), the probability distribution of the applied laser current *I* conditional on the observed velocity of the escape response **v** ≡ {*v*(*t*)}. Using Bayes’ theorem, we write

$$P\left(I|v\right)=\frac{P(v|I)P(I)}{P(v)}=\frac{1}{Z}P\left(v|I\right)P\left(I\right),$$ (1)

where *Z* is the normalization factor, and *P*(*I*) is the prior distribution of stimuli. When we characterize the velocity profiles of the noxious response of *C. elegans*, we notice that the worm can react to the stimulus in two different ways. Some worms pause after the heat stimulus, even at large laser currents (Fig 2B). These worms remain largely immobile for a few seconds, sometimes as long as the recording duration. Other worms actively reverse and follow the classic escape behavior (Fig 1). We choose to separate the active vs. the paused worms with a cutoff of 10 pixels/s, where 50 pixels is about one body length of the worm. To account for this heterogeneity in the behavior, we introduce the state variable *s*, which can take one of two values, *a* or *p*, for each individual worm. Then

P(v|I)=P(v|s=p,I)P(s=p|I)+P(v|s=a,I)P(s=a|I). (2)

We model the probability of the paused state *P*(*p*|*I*) by a sigmoid function (Fig 2C),

$$P\left(s=p|I,I_{0}\right)=\frac{1}{1+{\left(I/I_{0}\right)}^{2}},$$ (3)

where $I_{0}$ is the *pause current* threshold. Then the probability of the active state is

$$P\left(s=a|I,I_{0}\right)=1-P\left(s=p|I,I_{0}\right)=\frac{{\left(I/I_{0}\right)}^{2}}{1+{\left(I/I_{0}\right)}^{2}},$$ (4)

We infer $I_{0}$ from data by maximizing $\prod_{i=1}^{N_{\text{type}}}P\left(s_{i}|I_{i},I_{0}\right)$, where *N*_type_ is the number of trials with worms of the analyzed type, and *I*_*i*_ is the actual laser current for a particular trial. Note that each of the three data sets has its own pause current (25.9 ± 2.8, 26.6 ± 1.9, and 51.6 ± 6.3 mA for the control, ibuprofen, and mutant worms, respectively). Changes in this threshold, like that for the mutant, will result in different numbers of worms responding to the same stimulus, which can be consistent with the changes in the stimulus level, depending on whether the response profiles themselves stay stable. This is what we investigate next. Parenthetically, we note that the fraction of active worms is essentially the same as the percentage of the escape response, which has been used previously to quantify worm nociception [21]. Here we go further and additionally analyze the behavioral profiles of the responding worms.

*C. elegans* locomotion consists of a series of stereotyped postures and behavioral states [40, 41]. Further, in other animals, escape responses are stereotyped as well [6]. Therefore, it is natural to explore if the escape response of *C. elegans* is also stereotyped, separately for the paused and the active states. For paused worms, the escape velocity is small and independent of the laser current, and we model it as a multivariate normal variable,

$$P\left(v|p,I\right)=\frac{1}{{\left(2\pi \right)}^{\frac{T}{2}}{\left|\Sigma_{p}\right|}^{\frac{1}{2}}}\exp\left[-\frac{1}{2}{\left(v-u_{p}\right)}^{T}\Sigma_{p}^{-1}\left(v-u_{p}\right)\right],$$ (5)

where **u**_*p*_ is the mean velocity profile of the paused worms measured from data, which we call the *paused template* velocity. *Σ*_*p*_ is the empirical covariance of the paused velocity, and *T* is the total number of effectively independent time points in the velocity profile time series, determined using the autocorrelation structure of the profile (Materials and Methods).

We expect that, in the active state, the worm escape is laser current dependent. Specifically, we seek to represent it by a current-dependent rescaling of a stereotypical escape velocity, **v** ∼ *f*(*I*)**u**_*a*_, where *f* is the scaling function, and **u**_*a*_ is the *active template* velocity. Since various features of the worm escapes (the maximum reverse speed, the maximum reverse acceleration, and the time to the omega turn), scale non-linearly and saturate with the laser current (Fig 2A), the rescaling, *f*(*I*), must be sigmoidal. Further, some worms have nonzero velocities even at zero laser current, so that *f*(0) may be nonzero. Finally, the overall scale of the template can be absorbed in the definition of **u**_*a*_. The simplest scaling function obeying these constraints has only two parameters

$$f\left(I\right)\equiv f_{I_{1},I_{2}}\left(I\right)=I_{1}+\frac{I}{1+I/I_{2}},$$ (6)

where $I_{1}$ and $I_{2}$ are again constants, different for the three different worm types. With this, we write the probability of a velocity profile given the laser current *I* for the worm in an active state as a multivariate normal distribution

$$P\left(v|a,I\right)=\frac{1}{{\left(2\pi \right)}^{\frac{T}{2}}{\left|\Sigma_{a}\right|}^{\frac{1}{2}}}\times \exp\left[-\frac{1}{2}{\left(v-f_{I_{1},I_{2}}\left(I\right)u_{a}\right)}^{T}\Sigma_{a}^{-1}\left(v-f_{I_{1},I_{2}}\left(I\right)u_{a}\right)\right],$$ (7)

where *Σ*_*a*_ is the covariance of the average velocity profile. We find the constants $I_{1}$ and $I_{2}$, **u**_*a*_ and *Σ*_*a*_ by maximizing the likelihood of the observed data (Materials and Methods).

In summary, the probability of a velocity profile given the laser current in a certain trial is

$$\begin{array}{c} P\left(v|I\right)=\left\{\frac{1}{1+{\left(I/I_{0}\right)}^{2}}\times \frac{1}{{\left(2\pi \right)}^{\frac{T}{2}}{\left|\Sigma_{p}\right|}^{\frac{1}{2}}}\exp\left[-\frac{1}{2}{\left(v-u_{p}\right)}^{T}\Sigma_{p}^{-1}\left(v-u_{p}\right)\right]\right\}\\ +\left\{\frac{{\left(I/I_{0}\right)}^{2}}{1+{\left(I/I_{0}\right)}^{2}}\times \frac{1}{{\left(2\pi \right)}^{\frac{T}{2}}{\left|\Sigma_{a}\right|}^{\frac{1}{2}}}\exp\left[-\frac{1}{2}{\left(v-f_{I_{1},I_{2}}\left(I\right)u_{a}\right)}^{T}\Sigma_{a}^{-1}\left(v-f_{I_{1},I_{2}}\left(I\right)u_{a}\right)\right]\right\}. \end{array}$$ (8)

The overall model of the experiment, Eq (1), also includes *P*(*I*). To a large extent, this is controlled by the experimentalist, and details are described in *Materials and Methods*.

### Is the heat-evoked escape stereotyped?

The model we built assumes a stereotypical escape behavior. Is this assumption justified? Velocities in the paused state are very small (worms barely move). Thus whether the stereotypy assumption provides a good model of the data is determined largely by the stereotypy of the active worms. If the active stereotypical response template exists, then it should be possible to collapse the average velocity profiles onto a single curve by the following transformation

$$v_{\text{collapse}}=\frac{v_{a}}{f_{I_{1},I_{2}}\left(I\right)}.$$ (9)

**Fig 3:**
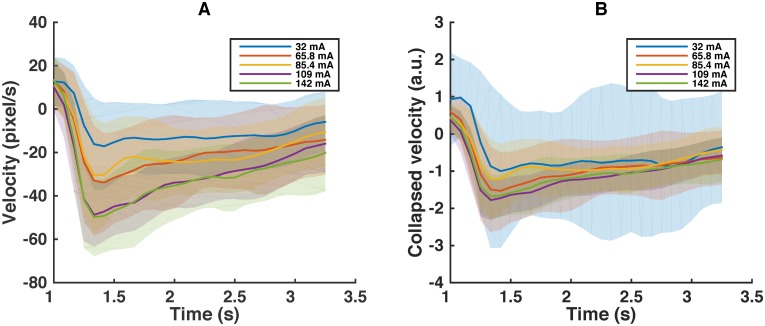
Collapse of the response behavior. (A) The mean (dark lines) and standard deviation (similar semitransparent light colors) of velocities of active control worms, binned for presentation purposes into five different groups of 40 worms each, based on the stimulus current. (B) The mean and standard deviation of velocities (same color code) in the same time period rescaled by $f_{I_{1},I_{2}}^{-1}\left(I_{i}\right)$. Rescaled mean velocities nearly collapse (see Fig 5 for quantification of the collapse). Note that the parameters $I_{1}$ and $I_{2}$ are optimized as in *Materials and methods* to collapse individual profiles, and not the five mean profiles illustrated here.

**Fig 4:**
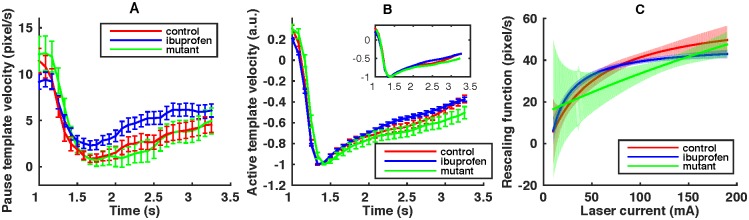
Stereotypical active response. (A) Paused template velocities. Error bars show the standard deviation of the template. Models are estimated separately for the three different worm types, indicated by different colors. Time *t* = 1 s corresponds to the moment of the stimulus application. (B) Normalized active template velocity. Each template velocity is normalized by the absolute value of its minimum | min_*t*_ **v**_*a*_ |. The error bars represent the model standard deviation, estimated by bootstrapping (Materials and Methods). The subfigure shows the template velocities adjusted by the time of maximum reverse velocity, illustrating that the three templates nearly match. (C) The rescaling function $f_{I_{1},I_{2}}\left(I\right)$ for the three different worm types, normalized by multiplying by the absolute value of the minimum of the active template velocity | min_*t*_ **v**_*a*_ |. The optimized parameter values are $I_{1}=-4.5,-4.5,66.5$ and $I_{2}=45.0,12.0,\infty$ for the control, ibuprofen, and mutant worms, respectively. We deliberately do not report error bars on individual parameters, but rather show the one standard deviation confidence intervals for the entire rescaling curve as shaded regions on the figure. The confidence region was again estimated using bootstrapping.

Indeed, the means of different bins collapse relatively compactly, providing evidence for the existence of the stereotypy in active responses (Fig 3). We show the template velocities and the non-linear scaling function *f* inferred from the control, ibuprofen, and mutant in Fig 4.

Note that the three active template velocity profiles are very similar (Fig 4A), but the mutant template velocity profile shows a response lag of 83 ms (one time frame at 12 Hz) compared to the control or ibuprofen data set. In other words, the pharmacological treatments and the mutations weakly affect the templated response, and the mutation slightly delays it. This bodes well for the assumption of the stereotypical response, definitely for ibuprofen and, to a somewhat lesser extent, for the mutant.

**Fig 5:**
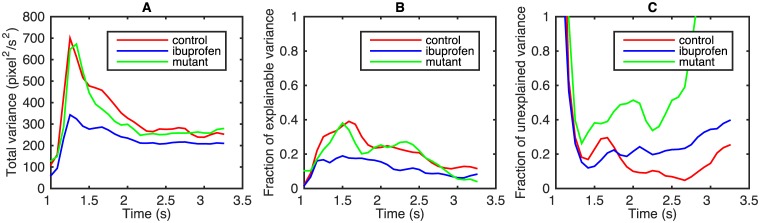
Variability of the active response. For the three worm types, in (A) we plot the total variance of velocity profiles $\sigma_{\text{total}}^{2}$. Note that these numbers depend strongly on the preprocessing of the data—in particular, smoothing of the velocity (with the 500 ms filter, Materials and Methods) decreases the total variance. In (B) we show the fraction of potentially explainable variance $\sigma_{I}^{2}/\sigma_{\text{total}}^{2}$. Finally, (C) shows the part of the explainable variance that was not explained by our statistical model $\sigma_{\text{res}}^{2}/\sigma_{I}^{2}$. See the main text for the discussion of the differences between the three worm types on this plot.

While the existence of the stereotypical patterns and their similarity across treatments is encouraging, we still need to quantify how good the statistical models are. In the ideal case, the variance $\sigma_{\text{collapse}}^{2}$ of the collapsed velocities, Eq (9), calculated over individual trials, would be zero. However, there are a number of expected sources of variance in the velocity, such as the individual variability and the model inaccuracies. To establish how good the stereotypical model fits are, we need to disambiguate these contributions. For this, we again partition all velocity profiles into five current bins. We then write the total variance of all responses as

$$\sigma_{\text{total}}^{2}=\sigma_{I}^{2}+\sigma_{\text{ind}}^{2},$$ (10)

where $\sigma_{\text{ind}}^{2}$ is the variance due to individual responses within each bin, and $\sigma_{I}^{2}$ is the current-driven variance of the mean responses across the bins. Since the individuality of the worms is not accounted for in our model, $\sigma_{I}^{2}$ represents the maximum potentially explainable variance in the data. The stereotypy-based model would be nearly perfect if $\sigma_{I}^{2}$ were to drop to zero after the *f*^−1^ rescaling. To explore this, we plot the total variance of the active response $\sigma_{\text{total}}^{2}$ (Fig 5A), and the fraction of the potentially explainable variance, $\sigma_{I}^{2}/\sigma_{\text{total}}^{2}$ (Fig 5B). The latter varies from 20% to 40% of the total variance, depending on the time post-stimulus and on the treatment. In both panels, the mutant and the control dataset are nearly indistinguishable, while the ibuprofen worms show a smaller variance, and a smaller fraction of the explainable variance. This is consistent with a smaller stimulus-driven response for this analgesic-like treatment. At the same time, these figures suggest that the decrease in the maximum reverse speed in the mutant worm (Fig 2A) should not be attributed entirely to the reduced perceived heat stimuli. Indeed, the similarity of the variance and the explainable variance in the control and the mutant worms, which have very different mean maximum reverse velocities, suggests the existence of an additional (explainable, non-templated) component in the response behavior of the mutant, which is not present in the control.

The explainable variance $\sigma_{I}^{2}$ is further split into the variance explained by the model, $\sigma_{m}^{2}$, and the residual variance, $\sigma_{\text{res}}^{2}$, which the model fails to explain:

$$\sigma_{I}^{2}=\sigma_{m}^{2}+\sigma_{\text{res}}^{2}.$$ (11)

In Fig 5C, we plot $\sigma_{\text{res}}^{2}/\sigma_{I}^{2}$, which is the fraction of the variance not captured by our model. Of the explainable variance, about 80% is captured by our model for the control and the ibuprofen worms in the window between 1 s and 3.3 s since the start of the trial, on average. This is a relatively large fraction for behavioral data, and provides an additional validation for our choice of a stereotypy-based model for representing escape behavior in these worms. Stability of the template itself between these two conditions, and the stability of the fraction of the explained variance suggest that much of the effect of ibuprofen can be attributed to the scaling of the templated response (and also the fraction of active worms). In other words, ibuprofen decreases the sensed heat stimuli.

In contrast, the unexplained variance for the mutant is about twice as large as that for the control, and approaches 100% at *t* > 2.7 s. This again illustrates that the templated response model is not very good for this treatment. Thus the mutations introduce changes in the behavior that are not consistent with a simple rescaling—mutations affect the fine motor behavior in addition to the sensory system per se.

### Using the statistical model

One of the goals of our study is to develop methods for quantitative assessment of the efficacy of pharmacological interventions to decreases sensed heat stimuli, at least in those cases where their action can be specifically interpreted as a change in thermal sensory transduction. We can use the developed statistical model for this. Specifically, taking the model derived from the control worms, we can infer the laser current from the behavior of all three different worm types. To the extent that the current inferred for the treated worms is smaller than that for the control worms at the same applied current, the heat stimuli level perceived by the treated worms is smaller.

**Fig 6:**
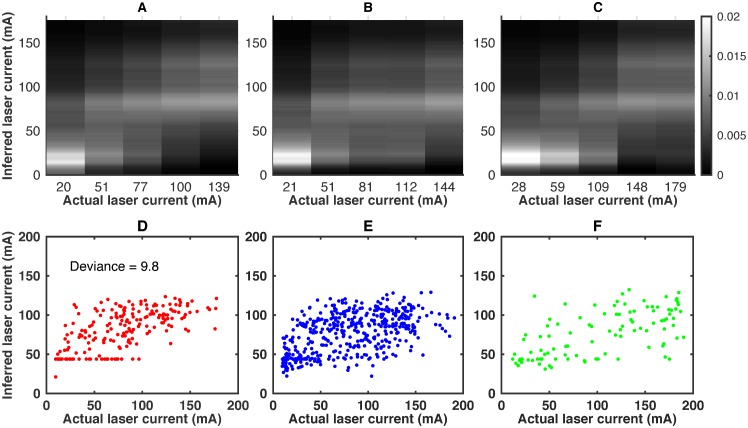
Inferring the perceived stimulus level from *C. elegans* escape behavior. The first row shows the conditional distributions of the inferred current vs. the actual applied current, partitioned into five bins, *P*_control_(*I*_inf_|*I*_*μ*_). Inference is done with the model of the control worm behavior. Panels (A), (B), (C) show the averaged probability of inferred laser current for the control, ibuprofen, and mutant worms, respectively. The second row shows the expected inferred laser current for each trial, ${\bar{I}}_{\text{inf},i}=\sum I_{\text{inf},i}P\left(I_{\text{inf},i}|v_{i}\right)$ vs. the applied current *I*_*i*_. The inference is again done using the control model, and panels (D), (E), (F) show the three worm types. The panel (D), (E), (F) is different from (A), (B), (C) since the first three show the average probability of inferred laser current while last three show the individual expected value of inferred laser current.

Fig 6 shows the overall structure of the inference done with the model. In the first row, we plot the conditional distribution of the inferred laser current given the actual applied current *I* for the three worm types, *P*(*I*_inf_|*I*, type). We again bin the trials using *I*_*i*_ into five bins *I*_*μ*_, *μ* = 1, …, 5 as before, and plot

$$P\left(I_{\text{inf}}|I_{\mu },\text{type}\right)=\sum_{i}^{N_{\text{type}}}P_{\text{control}}\left(I_{\text{inf}}|v_{i}\right)P_{\text{type}}\left(v_{i}|I_{\mu }\right).$$ (12)

Here *P*_type_(**v**_*i*_|*I*_*μ*_) is 1 if the stimulus on the *i*’th trial for this worm type was in the *I*_*μ*_ bin, and zero otherwise. Further, *P*_control_(*I*_inf_|**v**_*i*_) is given by the full model, Eq (8), with the parameters inferred for the control worm, and with the empirically observed velocities **v** in trial *i* for each worm type. We see that there is more probability concentrated at small *I*_inf_ for the ibuprofen and the mutant worms, suggesting a reduction in the perceived stimulus level. Similarly, in the second row in Fig 6, we plot the expected value, ${\bar{I}}_{i}$, of the distribution of the current inferred using the control model, *P*_control_(*I*_inf_|**v**_*i*_), for each of the individual trials in each of the three worm types. To the extent that the values for the ibuprofen and the mutant worms are again somewhat lower than for the control worms, there is some reduction in the perceived current by this measure as well.

However, these population averaged results wash out important differences in the structure of the stimulus-response relationship. To quantify these small effects more accurately, we now look at the perceived stimulus changes for individual worms in the datasets. Specifically, for each trial *i* in the control dataset, a trial *j*(*i*) with the closest value of the applied laser current is found in the ibuprofen / mutant dataset (the mean magnitude of the current mismatch is <1 mA for both the ibuprofen and the mutant worms). We then use the control model to calculate the expected value of the inferred current for the *j*th trial in the ibuprofen / mutant datasets. This expectation is subtracted from the expectation value of the inferred current for the matched trial *i* for the control dataset. The difference of the expectation values, averaged over all control worms, is our measure of the reduction in the perceived stimulus level

$$\Delta I_{\text{type}}=\frac{1}{N_{\text{control}}}\sum_{i}^{N_{\text{control}}}\left(I_{i,\text{control}}-I_{j(i),\text{type}}\right).$$ (13)

**Fig 7:**
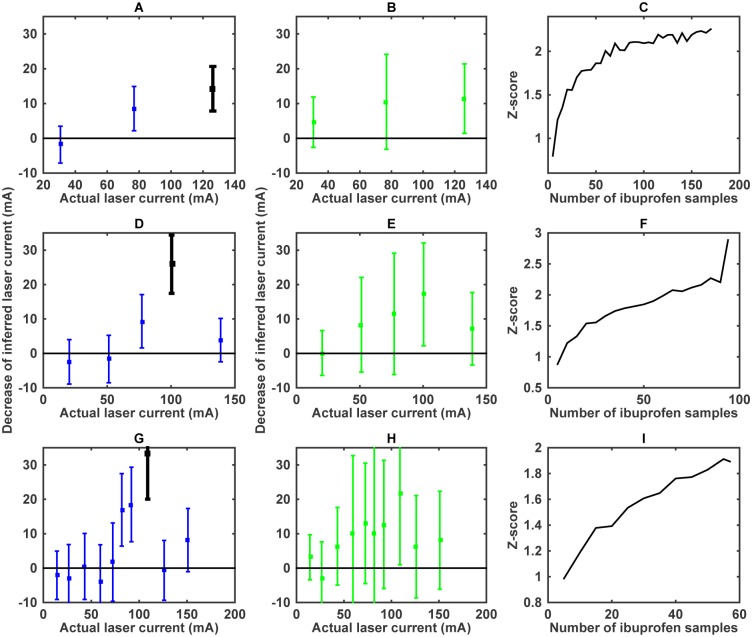
Perceived stimulus level decrease due to treatments. (A) Differences of the inferred laser current between the control and the ibuprofen datasets, Δ*I*_ibuprofen_. Errors represent standard deviations, estimated with bootstrapping. (B) Similar differences for the mutant worm, Δ*I*_mutant_. Error bars are estimated by bootstrapping. (C) Dependence of the statistical significance of the perceived stimulus level reduction (measured by the *Z* score) for ibuprofen at 110 mA on the number of ibuprofen trials.

We evaluate Δ*I*_type_ for different worm types and for control worms binned into the five usual current bins (Fig 7). To estimate the error of Δ*I*_type_, we bootstrap the whole analysis pipeline, see Materials and Methods. There is a statistically significant difference in stimulus perception between the ibuprofen and the control worms. The difference is most significant when the actual laser current is around 100–110 mA. This coincide with our observation (Fig 2A) that the most sensitive region of maximum reverse speed is around 100mA. Indeed, at smaller currents, the perceived stimulus level is small, many worms pause, and the behavior cannot be used to reliably estimate the stimulus level. At high current, the heat perception saturates, and all worms behave similarly, again reducing the ability to disambiguate the applied current level.

This analysis of ibuprofen worms achieves one of our main goals. It proves our ability to reconstruct stimulus from the behavior, and shows that analgesic-like effects of pharmacological perturbations can be quantified from the behavior. At the same time, Δ*I*_mutant_ turns out to be insignificant (Fig 7B), even though a large statistically significant difference exists between the mutant and the control behaviors (Fig 2A). This failure to detect a significant perceived stimulus reduction is because the templated response model is not very good for the mutant worm; thus our analysis cannot reliably assign a mutant trajectory on a given trial to a specific stimulus level. In other words, the large error bars in Fig 7B serve as yet another check for self consistency: effects of the mutations cannot be attributed just to changes in stimulus perception.

### Designing experiments: How many worms?

We expect our analysis to be useable for screening large numbers of chemicals for analgesic-like action. Since our approach targets one individual worm at a time, we need to estimate the number of worms needed to achieve statistical significance in such screening experiments. For this, we fix the number of control worms, arguing that these must be only analyzed once, and hence a relatively large number of them can be tested. We then focus on ibuprofen, whose action is analgesic-like in our experiments, and on the bin at 110 mA, where the worms experience the most significant perceived stimulus reduction. There are *N*_ibuprofen,110_ = 94 worms in this bin. We randomly sample with replacements *n* < *N*_ibuprofen,110_ worms from among these ibuprofen-treated worms and repeat our analysis pipeline, estimating the Δ*I*_ibuprofen_(*n*). Resampling 1000 times (both the ibuprofen and the control datasets), we also estimate the variance of Δ*I*_ibuprofen_(*n*), and hence the *Z* score as a function of *n* (Fig 7C). The plot here is an *underestimate* of the true *Z* score since resampling with replacements removes some stimulus values from the dataset, hence increasing the mismatch between control worms and the paired treated worms. Even with this, *Z* ≈ 2 is achieved at *n* ≈ 60 ibuprofen worms. In other words, in a typical screening experiment, one would need to test 200 or more worms to build the control model, and then at least ∼60 worms additionally for each treatment condition.

## Discussion

Typically a goal of sensory-response experiments is to develop a model that can predict the behavior in response to the stimuli. Here we wanted to do this in reverse. Our *grand goal* was to build a statistical model of the heat stimulus from careful measurements of the escape behavior of *C. elegans*, and to use this model to infer the changes in the perceived level of the stimulus felt by the organism due to perturbation in the sensory transduction pathway. Given this model, we could then measure changes in stimulus perception due to effects of chemicals and mutations, and use this as a basis to study the mechanism of sensory transduction in a genetically tractable organism amenable to high-throughput screens. As a representative data set, we choose to study the standard laboratory *C. elegans* strain N2, N2 treated with ibuprofen, and a mutant with defects in TRPV function. Other chemicals are also studied, but only ibuprofen is selected due to two reason. First, we selected chemicals that did not affect normal motion without laser stimulus, to make it more likely that the stereotypical behavior was not affected. Second, we made sure that the worm nonetheless displayed visually different behavior after the laser stimulus compared to N2 strain. Only ibuprofen passed these tests.

For the model to be successful, we had to meet a number of challenges. Since the worm could not communicate its perceived stimulus level to us directly we had to infer this level by reading the “body language” of the worm’s escape response. The difficulty with quantifying a behavioral response as a measure of perceived stimulus level is that drugs or mutations can affect locomotory behavior in addition to perturbing sensory transduction. So in an attempt to deconvolve these effects, we used the entire behavioral profile instead of making ad hoc measurements. We leveraged the fact that escape responses in *C. elegans* turn out to be highly stereotyped, so that the escape response can be modeled with a velocity profile template that scales non-linearly in response to an applied laser current. The success of the template in modeling the stereotyped wild-type escape response was confirmed by a functional collapse of the velocity profiles across different perceived stimulus levels. This discovery of invariance is important since it not only allowed us to effectively correlate escape behavior to the stimulus level, but it also allowed us to determine if the locomotory changes in our assay were due to changes specifically in the sensory transduction pathway or due to other general locomotory factors. By carefully accounting for the variation in our data and quantifying how much of this variation is captured by the model, we showed that the stereotypical behavior is unaffected by ibuprofen, save for changing the amplitude of the response. Thus this drug application likely reduced the perceived stimulus level in the worm. In contrast, a TRPV mutation changes locomotion in a way that is not as well captured by the template model. Thus we can be objectively critical about any inference made with this strain.

The model was also useful in determining key experimental parameters for future measurements. After verification that the model works well with the native and ibuprofen treated stimulus-response data, we quantified the changes in heat perception due to ibuprofen treatment. Our modeling and experimental assessment of escape behavior identified the optimal stimulus range and required number of trials to determine statistically significant differences between the inferred current of N2 in the untreated and treated conditions.

As a cautionary note, we point out that we avoid to call our heat stimulus as noxious, although the escape behavior of the worm in our experiment is similar to nociception. In the IASP definition, a nociceptive stimulus is an actually or potentially tissue-damaging event. The heat stimulus used in our experiment causes temperature increases of around 2°C in 0.1s, which does not have any evidence to damage the worm tissue. But previous research showed that the worm responses to small and rapid temperature increase in a nociception-like behavior [29]. Also a prolonged exposure to our heat stimulus is likely to cause damage to the worm tissue. Therefore although we are not calling our stimulus as noxious, we believe our model will be valid for nociceptive behavior.

Also we point out that many noxious responses, especially in larger animals, are not stereotyped (and hence less studied), and not all stereotyped behaviors are noxious responses. The stereotypy of escape in *C. elegans* has turned out to be helpful in disambiguating qualitatively different effects that ibuprofen and mutations have on nociception, and it is likely to be equally helpful in the future in characterizing effects of other mutations and perturbations. However, by itself the stereotypy should not be viewed as evidence for a nociceptive response, and neither should the absence of stereotypy be used as an evidence that a response is not noxious.

In conclusion, we have solved what we defined as a grand problem in stimulus-response quantification and built a general model that connects stereotyped behavior to stimulus in the context of *C. elegans* heat-induced escape responce. With a language to describe this relationship, it is now possible to study quantitatively the effects of genetics and chemicals on this sensorimotor behavior. We believe that the utility of the model is quite general and could be applied to different model systems. However, we particularly hope that this work helps further establish *C. elegans* as a model for nociceptive research.

## Materials and Methods

### Worm preparation and experiment design

All worms were grown and maintained under standard conditions [42], incubated with food at 20°C. Well fed worms were washed twice then gently spun down for 1 minute and the supernatant discarded by aspiration. We discovered empirically that ibuprofen affects the heat-induced escape response in our assay. For the drug application 100 *μ*L of ibuprofen in M9 at 100 *μ*M was added to the eppendorf tube. For the wild-type and mutant data set, M9 was used instead of the drug solution. Worms were then placed in an incubator for 30 minutes at 20°C. After that worms were poured onto a seeded agar plate and transferred to agar assay plates by a platinum wire pick. These assay plates were incubated at 20°C for 30 minutes, and then the experimental trials were done within the next 30 minutes. In total *N* = 201 worms for the control group, *N* = 441 worms for the ibuprofen group, and *N* = 100 worms for the mutant group (ocr-2(ak47) osm-9(ky10) IV; ocr-1(ak46)) group were tested. The mutant strain was obtained from the Caenorhabditis Genetics Center.

The heat stimulation instrument has been described previously [29]. In summary, an infrared laser is directed to heat the head of a freely crawling worm (∼0.5mm FWHM) on an agar plate. The laser pulse is generated with a randomly chosen laser current between 0 to 200 mA, with a duration of 0.1 s. The heating of the worm is nearly instantaneous, and it is directly proportional to the current, between 0 and 2°C for the current range used in our experiments. The temperature change at 60 mA current is 0.4°C ± 0.03°C, 100 mA current is 0.89°C ± 0.05°C and 150mA current is 1.4°C ± 0.2°C. Worms were stimulated only once and not reused. The movements of the worms are imaged using a standard stereomicroscope with video capture and laser control software written in LabVIEW. For each stimulus trial, a random worm is selected on the plate and its motion is sampled at 60 Hz for 15 s, and the laser is engaged 1 s after the start of the video recording.

### Data analysis

The recorded response videos were then processed with Matlab to calculate the time series of the worm centroid motion as described previously [29]. All the worms that were not stimulated near the head or were not moving forward in the beginning of the video were discarded. Numerical derivatives of the centroid times series were then taken and filtered with a custom 500 ms Gaussian filter, which was a one-sided Gaussian at the edges of the recorded time period, becoming a symmetric Gaussian away from the edges. This removed the noise due to numerical differentiation and also averaged out the spurious fluctuations in the forward velocity due to the imperfect sinusoidal shapes of the moving worm. We verified that different choices of the filter duration had little effect on the subsequent analysis pipeline. The direction of the velocity was determined by projecting the derivative of the centroid time series onto the head-to-tail vector for each worm, with the positive and negative velocity values denoting forward / backward motion, respectively.

The filtered velocity profiles needed to be subsampled additionally. This was because the statistical model of the data, Eq (8), involved covariance matrices of the active and paused velocity profiles, *Σ*_*p*_ and *Σ*_*a*_ (note that velocity profiles are not temporally translationally invariant due to the presence of the stimulus, thus the full covariance matrix is needed, and not a simpler correlation function). To have a full rank covariance matrix, the number of trials must be larger than the number of time points. Alternatively, regularization is needed for covariance calculation. The autocorrelation function for all three worm types showed a natural correlation time scale of ≳ 0.2 s, whether the data was pre-filtered or not. Thus subsampling at a frequency > 5 Hz would not result in data loss. Therefore, instead of an arbitrary regularization, we chose to subsample the data at 12 Hz, leaving us with 37 data points to characterize the first 3 s of the worm velocity trace after the stimulus application, 1 ≤ *t* ≤ 4 s since the start of the trial. Eq (8) additionally needs knowledge of *T*, the number of effectively independent velocity measurements in the profile. This is obtained by dividing the duration of the profile by the velocity correlation time. An uncertainty of such procedure has a minimal effect on the model of the experiment since it simply changes log likelihoods of models by the same factor, not changing which model has the maximum likelihood.

We then considered limiting the duration of the velocity profile used in model building: if velocities at certain time points do not contribute to the identification of *I*, they should be removed to decrease the number of unknowns in the model that must be determined from data (values of the templates at different time points). The first candidate for removal was the period of about 10 frames (0.16 s) after the laser stimulation since worms do not respond to the stimulus so quickly. However, removal of this time period had a negligible effect on the model performance, and we chose to leave it intact. In contrast, starting from 3.3 s (2.3 s after the stimulus) the fraction of explainable variance drops to nearly zero (Fig 5) since many worms already had turned by this time and resumed forward motion. Therefore, we eventually settled on the time in the 1.0…3.3 s range for building the model.

Whenever needed, we estimated the variance of our predictions by bootstrapping the whole analysis pipeline [43]. For this, we created 1000 different datasets by resampling with replacement from the original control dataset and the mutant / ibuprofen datasets. Control statistical models (the scaling function *f* and the velocity templates) were estimated for each resampled control dataset. Standard deviations of these models were used as estimates of error bars in Fig 4. For Fig 7, we additionally needed to form the closest control / treatment worm pairs. These were formed between the *resampled* data sets for all worm types as well. Standard deviations of Δ*I*_type_ evaluated by such resampling were then plotted in Fig 7 and used to estimate *Z* scores. Note that such resampling produces control / treatment paired worms that have slightly larger current differences than in the actual data; this leads to our error bars being *overestimates*.

Model in Eq (1) requires knowing *P*(*I*). In principle, this is controlled by the experimentalist, and thus should be known. In our experiments, *P*(*I*) was set to be uniform. However, as described above, some of the worms were discarded in preprocessing, and this resulted in non-uniformly distributed current samples. To account for this, we used the empirical *P*_emp_(*I*) in our analysis instead of *P*(*I*) = const. In turn, *P*_emp_(*I*) was inferred using a well-established algorithm for estimation of one-dimensional continuous probability distributions from data [44].

All of this analysis was implemented using Matlab, and the code is available for download from a public GitHub repository https://github.com/EmoryUniversityTheoreticalBiophysics/C.-elegans.

### Calculating the template velocities, the covariances, and the scaling function

The template for the paused state **u**_*p*_ is calculated by taking the average of all paused velocity profiles for each of the three worm datasets. The covariance *Σ*_*p*_ is then the covariance of the set of the paused velocity profiles.

For active worms, we start with fixed putative parameter values $I_{1}$ and $I_{2}$. We then calculate the active template **u**_*a*_ and the covariance matrix ∑_*a*_ by maximizing the likelihood in Eq (7)

$$\frac{\partial \sum_{i}^{N_{\text{type},a}}\log P\left(v_{i}|a,I_{i}\right)}{\partial u_{a}}\propto \sum_{i}^{N_{\text{type},a}}\left[v_{i}f_{I_{1},I_{2}}\left(I_{i}\right)-u_{a}f_{I_{1}\;I_{2}}^{2}\left(I_{i}\right)\right]=0,$$ (14)

$$\frac{\partial \sum_{i}^{N_{\text{type},a}}\log P\left(v_{i}|a,I_{i}\right)}{\partial \Sigma_{a}}\propto \sum_{i}^{N_{\text{type},a}}{\left[v_{i}-u_{a}f_{I_{1},I_{2}}\left(I_{i}\right)\right]}^{2}-{\left(\Sigma_{a}\right)}^{-1}=0,$$ (15)

where *N*_type,*a*_ is the number of active worms of the analyzed type. This gives:

$$u_{a}\left(I_{1},I_{2}\right)=\frac{\sum_{i=1}^{N_{\text{type},a}}v_{i}f_{I_{1},I_{2}}\left(I_{i}\right)}{\sum_{i=1}^{N_{\text{type},a}}f_{I_{1},I_{2}}^{2}\left(I_{i}\right)},$$ (16)

$$\Sigma_{a}=\sum_{i}^{N_{\text{type},a}}{\left[v_{i}-u_{a}f_{I_{1},I_{2}}\left(I_{i}\right)\right]}^{2}.$$ (17)

Having thus estimated **u**_*a*_ and *Σ*_*a*_ at fixed parameter values $I_{1}$, $I_{2}$, we maximize ∏_*i*_ *P*(**v**_*i*_|*a*,*I_i_*) over the parameters using standard optimization algorithms provided by MATLAB. We perform optimization from ten different initial conditions to increase the possibility that we find a global, rather than the local maximum.
